# Unigene-based RNA-seq provides insights on drought stress responses in *Marsdenia tenacissima*

**DOI:** 10.1371/journal.pone.0202848

**Published:** 2018-11-30

**Authors:** Heng-Ling Meng, Wei Zhang, Guang-Hui Zhang, Jian-Jun Wang, Zhen-Gui Meng, Guang-Qiang Long, Sheng-Chao Yang

**Affiliations:** 1 The Life Science and Technology College, Honghe University, Mengzi, Yunnan, People’s Republic of China; 2 Yunnan Research Center on Good Agricultural Practice for Dominant Chinese Medicinal Materials, Yunnan Agricultural University, Kunming,Yunnan, People’s Republic of China; Hainan University, CHINA

## Abstract

*Marsdenia tenacissima* is a well-known anti-cancer medicinal plant used in traditional Chinese medicine, which often grows on the karst landform and the water conservation capacity of land is very poorly and drought occurrences frequently. We found *M*. *tenacissima* has strong drought resistance because of continuousdrought16 d, the leaves of *M*. *tenacissima* were fully curly and dying. But the leaves were fully almost recovering after re-watering 24h. The activity of SOD and POD were almost doubled under drought stress. The content of osmotic regulating substance proline and soluble sugar were three times than control group. But after re-watering, these indexes were declined rapidly. Three cDNA libraries of control, drought stress, and re-watering treatments were constructed. There were 43,129,228, 47,116,844, and 42,815,454 clean reads with Q20 values of 98.06, 98.04, and 97.88respectively.SRA accession number of raw data was PRJNA498187 on NCBI. A total of 8672, 6043, and 6537 differentially expressed genes (DEGs) were identified in control vs drought stress, control vs re-watering, and drought stress vs re-watering, respectively. In addition, 1039, 1016, and 980 transcription factors (TFs) were identified, respectively. Among them, 363, 267, and 299 TFs were identified as DEGs in drought stress, re-watering, and drought stress and re-watering, respectively. These differentially expressed TFs mainly belonged to the bHLH, bZIP, C2H2, ERF, MYB, MYB-related, and NAC families. A comparative analysis found that 1174 genes were up-regulated and 2344 were down-regulated under drought stress and this pattern was the opposite to that found after re-watering. Among the up-regulated genes, 64 genes were homologous to known functional genes that directly protect plants against drought stress. Furthermore, 44 protein kinases and 38 TFs with opposite expression patterns under drought stress and re-watering were identified, which are possibly candidate regulators for drought stress resistance in *M*. *tenacissima*. Our study is the first to characterize the *M*. *tenacissima* transcriptome in response to drought stress, and will serve as a useful resource for future studies on the functions of candidate protein kinases and TFs involved in *M*. *tenacissima* drought stress resistance.

## Introduction

Drought is one of the most severe threats to crop production worldwide. It causes considerable yield losses and effects food security [1]. Global warming means that drought will occur more frequently and will affect crop production more severely [1–2]. Therefore, developing drought-tolerant crops is currently one of the main objectives of breeding programs. However, a deeper understanding of the molecular mechanisms underlying drought tolerance in crops is essential if new varieties with improved drought resistance are to be developed.

Over the last decade, the molecular mechanisms underlying plant drought tolerance have been widely investigated in different species using gene microarrays [3–7]. As a result, thousands of genes have been identified that respond to drought stress by changing their expression levels. Usually, these drought stress-inducible genes have been divided into two groups. One group that directly protects plants against drought stress are involved in water transport (aquaporin) [8–9], scavenging of free oxygen radicals (superoxide dismutase, catalase, and peroxidase), maintaining cellular membrane integrity (proline, mannitol, glycine, and betaine), and protecting macromolecules (chaperones and late embryogenesis abundant proteins) [10–11]. The second group is involved in signal perception, signal transduction, and amplification. These include receptor proteins, protein kinases, protein phosphatases, and transcription factors (TFs) [10–12]. To date, many drought stress-inducible genes, especially transcription factors, have been functionally demonstrated to play crucial roles in plant drought tolerance. These transcription factors include ABA-dependent MYC/MYB and WRKY, ABA-responsive element binding/ABA-binding factor (AREB/ABF), ABA-independent dehydration-responsive element-binding proteins (DREB), C-repeat/drought-responsive element (CRT/DRE), and NAC transcription factors [13–17].

RNA sequencing (RNA-seq) technology with higher specificity and sensitivity has emerged as a powerful technique for the detection of genes, transcripts, and differential expression profiling, especially, monitoring gene function at the entire genome level in a species without any available genome information [18]. To date, RNA-seq technology has been used to dissect the molecular responses of plant drought tolerance in many plants, especially in non-model plants without available genome information, and some new drought stress genes have been identified [19–24]. Although RNA-seq technology has led to major advances in understanding plant responses to drought, knowledge about the molecular mechanisms underlying drought tolerance in medicinal plants is still extremely limited.

*M*. *tenacissima* is a well-known anti-cancer medicinal plant used in traditional Chinese medicine. The anticancer injection developed by extracting the glycosides from *M*.*tenacissima* has a good market value and application prospect (about 800 million RMB a year). Furthermore, it also has function in treat asthma, tracheitis, tonsillitis, pharyngitis, cystitis, and pneumonia [25–27]. *M*. *tenacissima* is widely distributed in tropical to subtropical areas across Asia, particularly in Guizhou and Yunnan Provinces in China where are the karst landform and the water conservation capacity of land is very poorly and drought occurrences frequently [28]. Drought stress clearly negatively impacts the normal growth and development of *M*. *tenacissima*,that leads to yield losses and plant quality decline [29]. However, to date, the molecular mechanism controlling drought tolerance in *M*. *tenacissima* is unknown, and no drought tolerance gene has been identified. In this study, we performed a comprehensive transcriptome sequencing analysis to explore the drought-tolerance mechanism in *M*. *tenacissima* and to identify the candidate genes that could potentially be used to improve crop drought resistance.

## Materials and methods

### Plant material, growth conditions, and drought stress treatments

The *M*. *tenacissima* “Yunnan” was used in this study, which was supplied by Yunnan Xintong Plant Pharmaceutical Co., Ltd. (Mengzi,Yunnan,China). The *M*. *tenacissima* seeds were surface-sterilized in 0.5% (w/v) NaClO for 15 min. Then they were sown in pots filled with peat and vermiculite (v/v = 3:1), and left to germinate in a greenhouse at 25°C. The two-week-old *M*. *tenacissima* seedlings were individually transferred to a small flowerpot containing 1 kg soil (humus soil:garden soil = 1:1) and grown in an artificial climate incubator under natural drought stress treatment(12 h/12 day/night, light 4000 lx,temperature:23°C/16°C day/night, air relative humidity: 75%/55% day/night).

**Fig 1:**
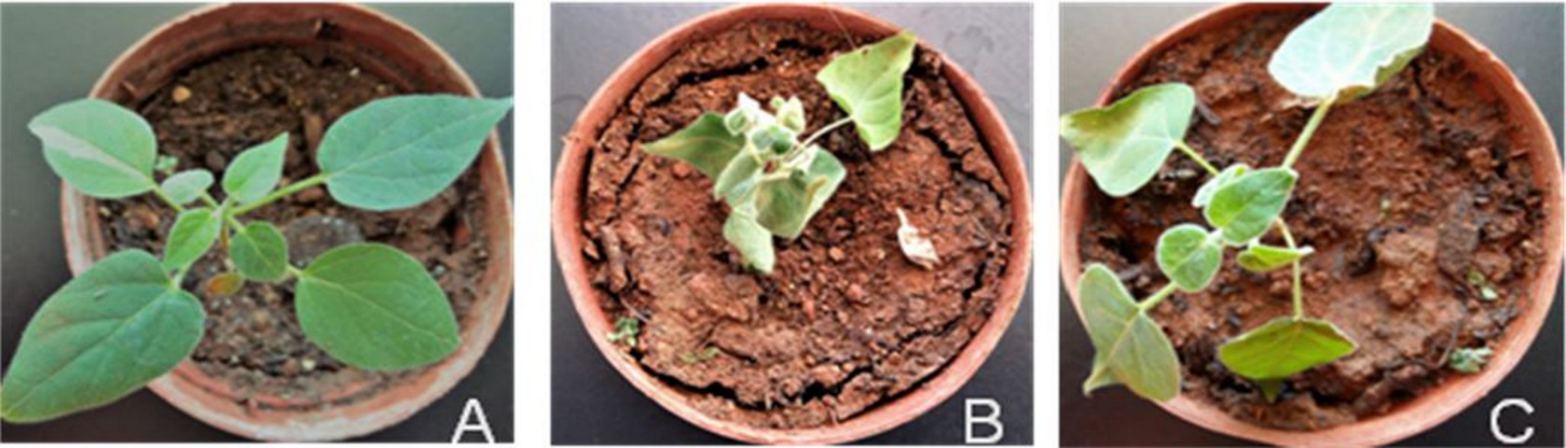
The phenotype of three groups of *M*. *tenacissima*.

In the drought treatment, 10–15-cm high plants were split into three groups with ten plants in each group. The control group of plants was supplied water every two days. The drought stress group of plants was not supplied water until all leaves were curing (about 16 days) (Fig 1B). The re-watering group of plants was not supplied water until the plant drought phenotype was the same as the drought stress group, then sample were taken when all curing leaves fully expanded (about 24 hours after watering) (Fig 1C).

### The determination of stress physiological indexes of *M*. *tenacissima*

The test materials were leaves of each group, using random sampling method. Each process was repeated three times. The contents of soluble sugar, malondialdehyde (MDA) and superoxide dismutase (SOD) were determined by anthrone method, thiobarbital colorimetry and nitroblue tetrazole photochemical reduction method respectively. The guaiacol method was used to measure the content of peroxidase (POD)[30].The experimental data were analyzed by single factor anova method of SPSS 21.0.

### Total RNA extraction and cDNA synthesis and sequencing

The roots, stems, and leaves from three randomly selected plants in each group were collected and stored at –80°C for RNA extraction. Total RNA was extracted from each sample using Triazol reagent (TaKaRa, Dalian, China) according to the manufacturer’s instructions. The samples were then treated with DNase I to remove any contaminated genomic DNA. The integrity and purity of the RNA was verified by an ultraviolet spectrophotometer (OD260/OD280 ratios of 1.89 to 2.08) and 1.2% agarose gel electrophoresis. The RNA from the roots, stems, and leaves of each group of plants was pooled. The cDNA libraries were then constructed according to Huang et al. [31]. The cDNA libraries were sequenced on a HiSeq2000 (Illumina, San Diego, CA, USA) according to the manufacturer’s standard protocols to generate 100-bp paired-end reads.

### Acquisition of clean reads and mapping

Raw reads from the cDNA library were filtered to remove low-quality reads and adaptors using the program FASTX-Tool kit (http://hannonlab.cshl.edu/fastx_toolkit/) to produce the clean reads. The clean reads were mapped to the reference transcriptome dataset (NCBISRA140234) using SOAP aligner/soap2 software (Li et al., 2009). The total mapped reads were kept for further analysis.

### Identification of differentially expressed genes (DEGs)

The DEGs between treatments were identified based on the Reads Per Kilobase per Million (RPKM) value calibrated by DEGseq [32–33]. Genes with a “q value < 0.005” and a “fold change |log2| > 1” were deemed to be significantly differentially expressed between the two samples.

### Functional annotation and classification

The DEGs were annotated using the following databases: the NR protein database (NCBI), Swiss Prot, Gene Ontology (GO), the Kyoto Encyclopedia of Genes and Genomes (KEGG) database, and the Clusters of Orthologous Groups database (COG) according to the methods of described by Zhou et al [34]. Pathways and GO function enrichment analyses were performed as previously described [35]. The transcription factor (TF) responses to drought stress were identified according to the method described by Zhao et al [36].

### Quantitative real-time PCR (qRT-PCR) verification of DEGs

To evaluate the accuracy and validity of the transcriptome sequencing data, 24 genes with differential expressions were selected to carry out the qRT-PCR analysis. Primers were designed using the BioXM 2.6 software, and the primer sequences are listed in S1 Table. The GAPDH (glyceraldehyde-3-phosphate dehydrogenase) gene was used as a reference gene. The qRT-PCR analysis of each gene was performed with three biological replicates according to the SYBR Premix ExTaq^TM^ protocol (TaKaRa) on a Light Cycler 480 Real-Time PCR machine (Roche Diagnostics Ltd., Switzerland). The relative expression level of each gene was calculated using the 2^-(ΔΔCt)^ method. The expression value of each gene from qRT-PCR and RNA-seq was log2 transformed so that the qRT-PCR data could be compared with the RNA-seq results.

## Results

### The analysis of physiological indexes in *M*. *tenacissima* of three groups

**Fig 2:**
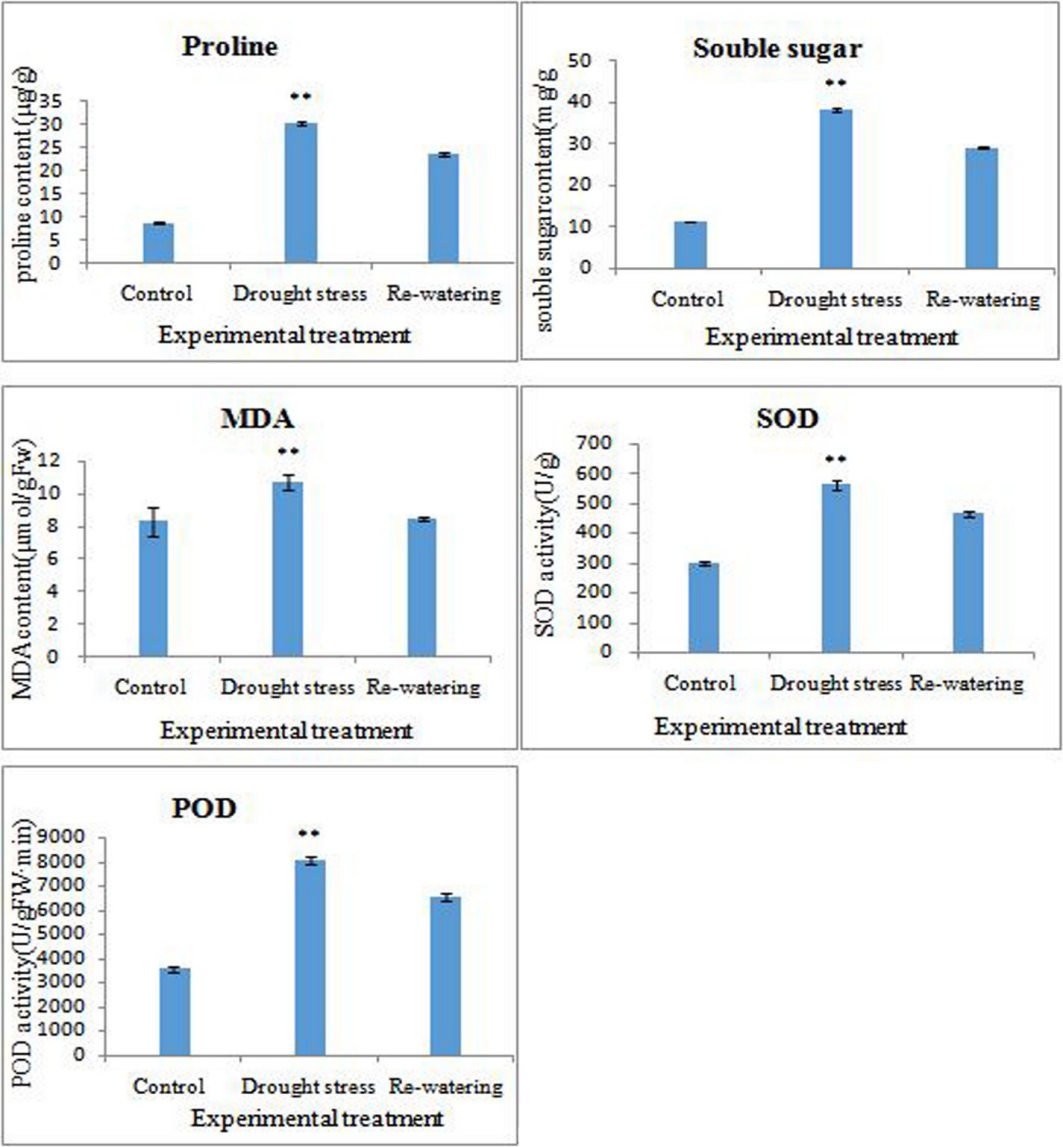
The change of 5 physiological indexes among control, drought stress and re-watering.

We measured some response indexes of abiotic stress, such as SOD, POD, MDA, etc. We found that the content of 5 indexes was significantly increasing under drought stress and 5 indexes were significantly decreasing after re-watering 24 hours. The re-watering group was significant higher than control group except MDA, which indicated that although morphologically restored, physiological stress has not completely relieved (Fig 2).

### Transcriptome sequencing, data statistics and evaluation, and reads mapping

To understand the drought-response molecular mechanism in *M*. *tenacissima* and identify potential candidate genes involved in drought tolerance, deep RNA sequencing of *M*. *tenacissima* seedlings subjected to drought and subsequent re-watering was performed using the Illumina sequencing platform. A total of 43,983,844, 48,059,552, and 43,744,500 raw reads were obtained from the control, drought stress, and re-watering cDNA libraries, respectively (Table 1).And SRA accession number was PRJNA498187 on NCBI. After removing the low-quality reads and adaptors, 43,129,228, 47,116,844, and 42,815,454 clean reads were produced, which accounted for 98.06%, 98.04%, and 97.88% of the raw reads, respectively. SRA accession number is PRJNA498187 on NCBI (Table 1). Furthermore, 32,879,580 (76.24%), 36,085,718 (76.59%), and 34,482,932 (80.54%) clean reads were mapped to the reference transcriptome (NCBI SRA140234) and 44,112, 39,307, and 39,608 genes were generated by SOAP aligner/soap2 software, respectively (Table 1). The total mapped reads were used to estimate the gene expression levels.

**Table 1:** Original data statistics.

Analysis reads	control	drought stress	re-watering
Raw reads	43,983,844	48,059,552	43,744,500
Clean reads	43,129,228	47,116,844	42,815,454
Clean bases (Gb)	5.02	5.48	4.98
Q20	98.06	98.04	97.88
Average length	859	985	975
Total mapped reads (%)	32,879,580 (76.24)	36,085,718 (76.59)	34,482,932 (80.54)
Unique mapped reads (%)	32,825,505 (76.11)	36,000,522 (76.41)	34,428,760 (80.41)
Gene number	44,112	39,306	39,607

### Identification of DEGs responding to drought stress

**Fig 3:**
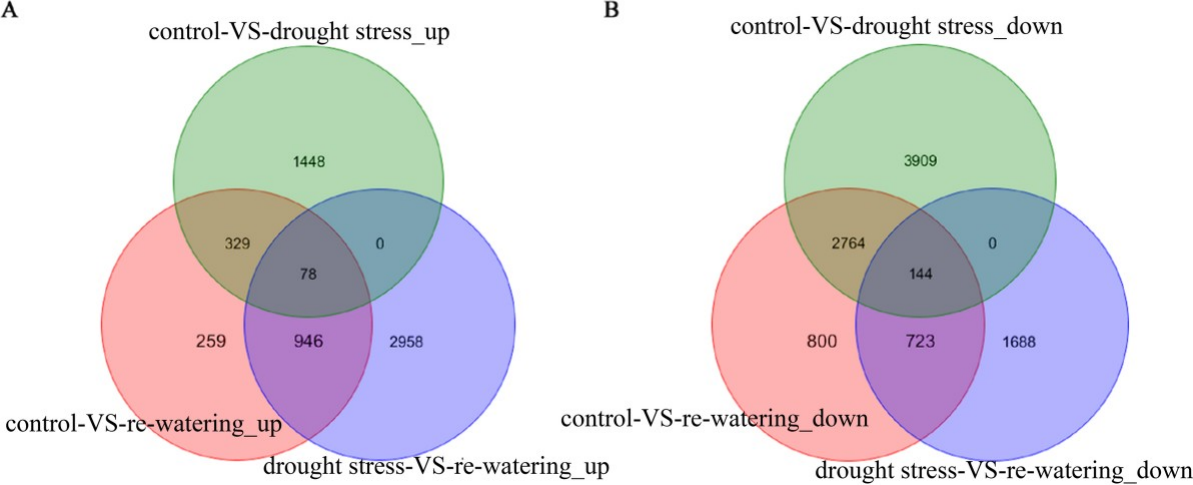
Venn diagram analysis of differentially expressed genes.

**Fig 4:**
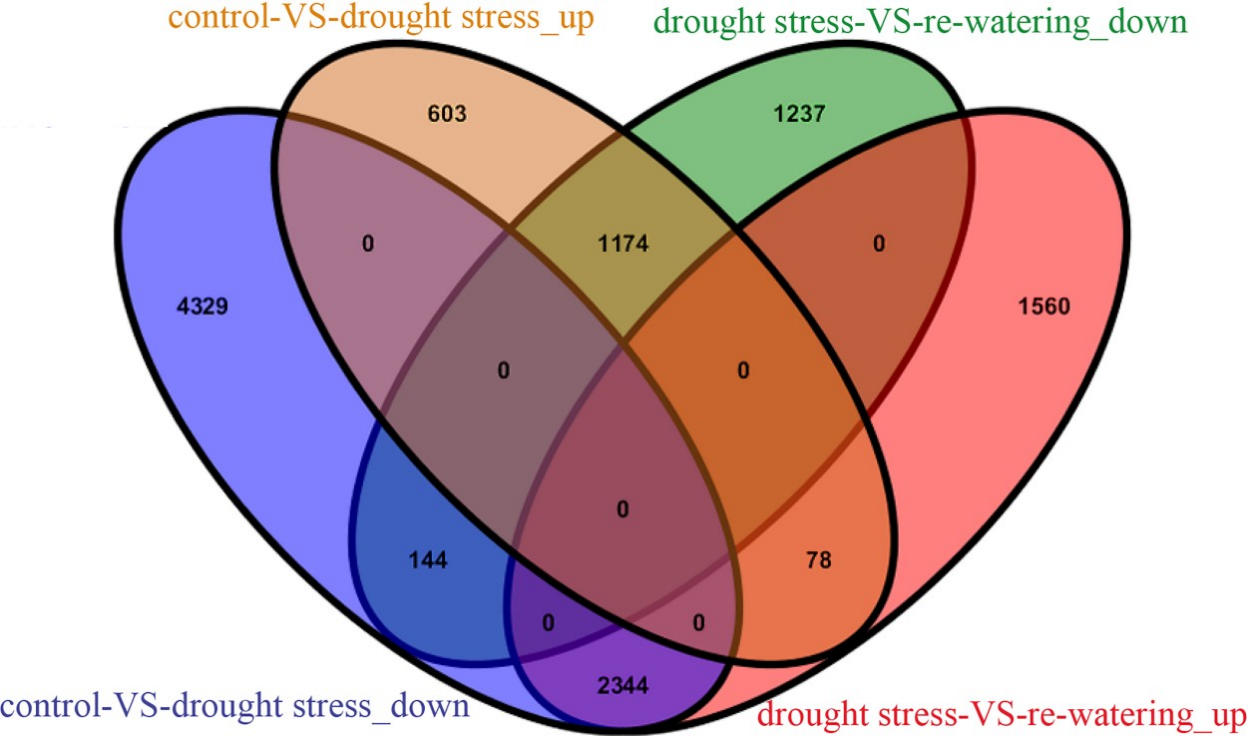
Venn diagram analyses of differentially expressed genes between control vs. drought stress, and drought stress vs.re-watering.

The genes from each treatment group were subjected to a pairwise comparison to identify the DEGs after using a blast algorithm with the preset cutoffs. As a result, a total of 21,254 DEGs were identified. A comparison between control and drought stress showed that 1855 genes were up-regulated, and 6817 genes were down-regulated. Between control and re-watering, 1612 genes were up-regulated and 4431 genes down-regulated. A further 3982 genes were found to be up-regulated, and 2555 genes were down-regulated between drought stress and re-watering, and 78 up-regulated genes and 144 down-regulated genes were identified in all three comparison groups (Fig 3). Notably, 1174 of the 1855 induced genes and 2344 of the 6817 repressed genes under drought stress were down-regulated, but were up-regulated after re-watering (Fig 4, S1 and S2 Tables). These genes probably play important roles in tolerance to drought stress.

### GO annotation of DEGs from the three comparison groups

**Fig 5:**
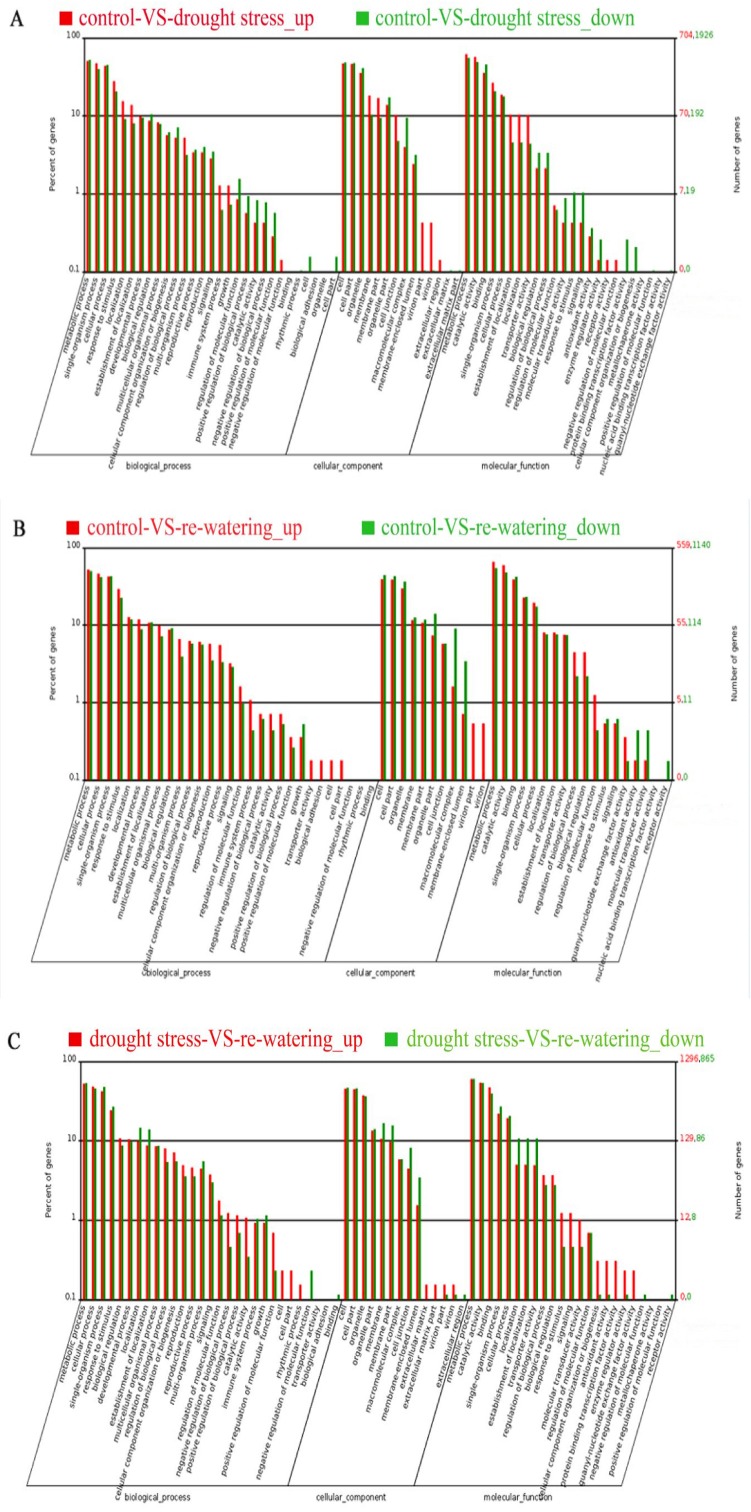
Gene Ontology (GO) categorization of the differentially expressed genes in the three comparison groups.

GO classification was performed to investigate the functions of the DEGs in the three comparison groups. After comparing control with drought stress, 2628 DEGs (704 up-regulated genes and 1924 down-regulated genes) were assigned to 67 main functional groups in the “biological processes”, “cellular components”, and “molecular functions” categories. When control was compared to re-watering, 1699 DEGs (559 up-regulated genes and 1140 down-regulated genes) could be functionally assigned to the relevant terms. The drought stress versus re-watering comparison functionally assigned 2161 DEGs (1296 up-regulated genes and 865 down-regulated genes) to the relevant terms. The top three significantly enriched GO functional annotation categories were “metabolic process”, “cell”, and “catalytic activity” (Fig 5).

### KEGG pathway analysis of DEGs

To determine whether these DEGs engaged in specific pathways, we performed a detailed KEGG pathway classification by searching against the KEGG pathway database. A total of 1910 of the DEGs from control vs. drought stress could be annotated into 120 pathways. The top five pathways were “metabolic pathways” (553), “biosynthesis of secondary metabolites” (229), “ribosome” (143), “starch and sucrose metabolism” (71), and “oxidative phosphorylation” (60) (S3 Table)_._ Between control vs. re-watering, a total of 1210 of the DEGs could be classified into 114 pathways.

The top five pathways were “metabolic pathways” (352), “biosynthesis of secondary metabolites” (205), “ribosome” (92), “plant hormone signal transduction” (47), and “oxidative phosphorylation” (47) (S4 Table)_._ For drought stress vs. re-watering, a total of 1426 of the DEGs could be annotated into 115 pathways. The top five pathways were “metabolic pathways” (457), “biosynthesis of secondary metabolites” (255), “ribosome” (65), “starch and sucrose metabolism” (63), and “plant hormone signal transduction” (58) (S5 Table)_._

### Transcription factor responses to drought stress and water stimulus

Transcription factors are known to play vital roles in plant abiotic stress tolerance because they can regulate the expression of numerous downstream genes. A total number of 1039, 1016, and 980 TFs were identified in control, drought stress, and re-watering, respectively (Table 2).

**Table 2:** Transcription factors (TFs) in the sequencing libraries.

TFs	control vs drought stress	control vs re-watering	drought stress vs re-watering
Total differentially expressed TFs	363	267	299
Up-regulated differentially expressed TFs	81	77	174
Down-regulated differentially expressed TFs	282	190	125

**Fig 6:**
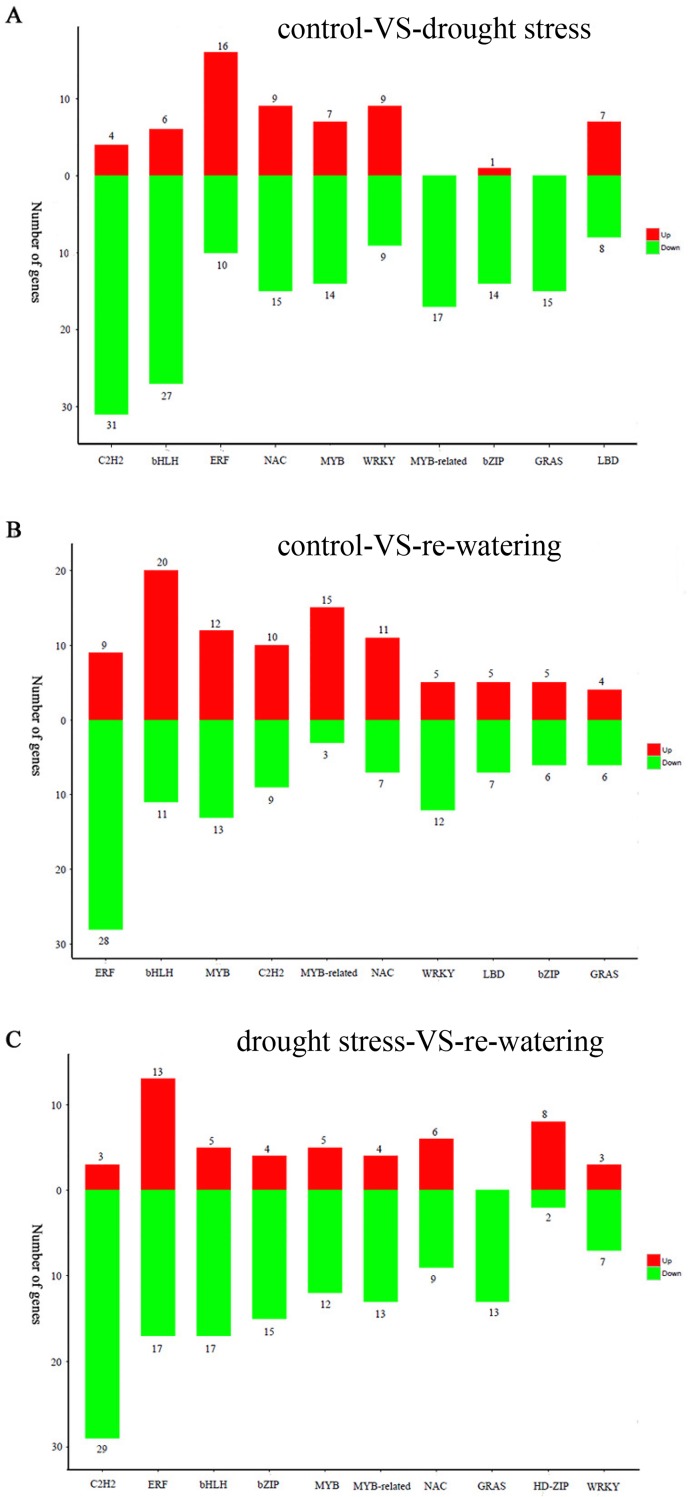
The top 10 families of differentially expressed transcription factors in the control vs. drought stress group (5A), the control vs. re-watering group (5B), and the drought stress vs. re-watering group (5C).

The number of TFs identified in the drought stress and re-watering library was slightly less than in control. In addition, 363, 267, and 299 TFs were identified as DEGs in control vs. drought stress, control vs. re-watering, and drought stress vs. re-watering, respectively. Further analysis revealed that the 363 DEGs from control vs. drought stress could be grouped into 42 families, and the top five families were C2H2 (35), bHLH (33), ERF (26), NAC (24), and MYB (21) (Fig 6A, S6 Table). Similarly, the 267 DEGs from control vs re-watering were grouped into 37 families, and most of the DEGs (103) belonged to the C2H2, ERF, bHLH, and bZIP families (Fig 6B, S7 Table). Furthermore, in the comparison between drought stress and re-watering, 299 DEGs were involved in a total of 43 TF families. Among these TF families, ERF (37), bHLH (31), MYB (25), C2H2 (19), and MYB-related (18) were the top five families with the most genes (Fig 6C, S8 Table).

A further analysis of the quantity relationship between up- and down-regulated TFs in the three comparison groups found that the number of up-regulated genes was significantly lower than the number of down-regulated genes in both control vs. drought stress and control vs. re-watering, but was the reverse in drought stress vs. re-watering (Table 2). In the control vs. drought stress comparison, the number of down-regulated TFs was 3-fold more than the up-regulated ones. The largest number of down-regulated TFs was found in the C2H2 family, while the ERF family contained the largest number of up-regulated TFs (Fig 6A). Similarly, in control vs. re-watering, the number of down-regulated TFs was 2-fold higher than the up-regulated ones. The ERF and bHLH families contained the largest number of down-regulated and up-regulated TFs, respectively (Fig 6B). In drought stress vs. re-watering, the number of down-regulated TFs was lower than the number of up-regulated TFs.

The C2H2 family contained the largest number of down-regulated TFs, whereas the ERF family had the largest number of up-regulated TFs (Fig 6C).

### Identification of candidate genes for drought stress resistance

To identify the candidate genes for drought stress resistance in *M*. *tenacissima*, 1174 genes that were induced by drought stress and repressed by re-watering were screened. A blast analysis showed that 855 of the 1174 genes had a functional description (S9 Table). Further analysis found that 64 genes were homologous to the known functional genes that directly protects plants against drought stress, which include aquaporin, late embryogenesis abundant protein, chaperone, dehydration responsive protein, pleiotropic drug resistance protein, alcohol dehydrogenase, peroxidase, proline metabolism genes, trehalose synthesis-related genes, flavonoid synthesis-related genes, mannitol transporter, sugar transporter, peptide transporter, MATE efflux protein, and ABC transporter genes (Table 3). In addition, histone, histone deacetylase, and methyltransferase were also found, which suggested that epigenetic regulation was involved in the *M*. *tenacissima* drought stress resistance mechanism (Table 3).

**Table 3:** The putative functional genes that were induced by drought stress and repressed by re-watering.

Gene ID	Fold change (drought stress/control)	Fold change (re-watering/drought stress)	Blast swissprot
**Unigene0008635**	3.1	-2.2	glutamate dehydrogenase A-like [Solanum lycopersicum] (Arginine and proline metabolism)
**Unigene0012721**	10.9	-4.8	trehalose-6-phosphate synthase [Camellia sinensis]
**Unigene0045759**	2.3	-4.2	probable trehalose-phosphate phosphatase J-like [Solanum lycopersicum]
**Unigene0022077**	8.5	-3.7	alpha-trehalose-phosphate synthase [UDP-forming] 9-like [Solanum lycopersicum]
**Unigene0027014**	2.1	-16.6	Late embryogenesis abundant hydroxyproline-rich glycofamily protein [Theobroma cacao]
**Unigene0009110**	11.9	-8.1	Late embryogenesis abundant hydroxyproline-rich glycofamily protein [Theobroma cacao]
**Unigene0013711**	3.8	-7.9	late embryogenesis abundant protein group 9 protein, partial [Genliseaaurea]
**Unigene0023521**	2.5	-3.3	aquaporin 1 [Nicotiana tabacum]
**Unigene0021777**	2.2	-3.0	aquaporin protein AQU20 [Camellia sinensis]
**Unigene0009422**	14.5	-15.4	dehydration responsive protein [Corchorusolitorius]
**Unigene0019806**	2.3	-5.7	dehydration responsive protein [Corchorusolitorius]
**Unigene0020350**	2.0	-9.6	probable mitochondrial chaperone BCS1-B-like [Fragariavesca subsp. vesca]
**Unigene0016740**	4.7	-7.0	chaperone protein dnaJ 11, chloroplastic-like [Vitisvinifera]
**Unigene0012659**	3.1	-2.2	Heat shock protein 70 (Hsp 70) family protein [Theobroma cacao]
**Unigene0016751**	2.5	-2.4	Heat shock protein DnaJ with tetratricopeptide repeat isoform 1 [Theobroma cacao]
**Unigene0008874**	3.1	-4.1	copper chaperone [Populus alba x Populusglandulosa]
**Unigene0022185**	2.8	-2.2	pleiotropic drug resistance protein 1-like [Vitisvinifera]
**Unigene0024248**	13.8	-11.5	pleiotropic drug resistance protein 2-like [Vitisvinifera]
**Unigene0026840**	3.2	-2.5	pleiotropic drug resistance protein 2-like [Solanum lycopersicum]
**Unigene0010544**	5.2	-5.1	alcohol dehydrogenase-like protein [Ocimumbasilicum]
**Unigene0014656**	2.2	-2.7	alcohol dehydrogenase [Solanum tuberosum]
**Unigene0032109**	25.4	-22.2	cinnamyl alcohol dehydrogenase [Neolamarckiacadamba]
**Unigene0021885**	158.8	-49.6	sinapyl alcohol dehydrogenase-like 3 [Nicotiana tabacum]
**Unigene0026625**	2.1	-5.5	peroxidase 4 [Vitisvinifera]
**Unigene0007407**	4.8	-12.5	peroxidase 10-like [Solanum lycopersicum]
**Unigene0037539**	6.4	-4.5	peroxidase [Populus alba x Populusglandulosa]
**Unigene0020714**	3.0	-4.4	peroxidase 4 [Litchi chinensis]
**Unigene0008948**	5.2	-4.6	peroxidase 25 [Vitisvinifera]
**Unigene0045735**	3.8	-6.1	peroxidase 73-like [Solanum lycopersicum]
**Unigene0023720**	3.2	-4.3	phenylalanine ammonia lyase [Catharanthusroseus]
**Unigene0023719**	3.8	-3.4	phenylalanine ammonia lyase [Catharanthusroseus]
**Unigene0022294**	3.0	-2.7	hydroxycinnamoyl-CoA quinatehydroxycinnamoyltransferase [Coffea canephora]
**Unigene0021581**	12.1	-2.6	flavonoid 3'-monooxygenase-like [Solanum lycopersicum]
**Unigene0017542**	2.4	-2.1	hydroxycinnamoyl-CoA shikimate/quinatehydroxycinnamoyltransferase [Coffea canephora]
**Unigene0018052**	5.0	-6.3	mannitol transporter [Artemisia annua]
**Unigene0023659**	2.1	-2.9	mannitol transporter [Artemisia annua]
**Unigene0019188**	2.7	-2.6	sugar transport protein [Coffea canephora]
**Unigene0023657**	3.7	-2.5	sugar transport protein [Coffea canephora]
**Unigene0021497**	8.1	-55	sugar transporter ERD6-like 7-like [Vitisvinifera]
**Unigene0014067**	3.6	-6.5	sugar transport protein 14-like [Solanum lycopersicum]
**Unigene0036310**	16.1	-17.0	bidirectional sugar transporter SWEET16-like [Solanum lycopersicum]
**Unigene0041988**	21.5	-45.1	sugar transporter ERD6-like 16-like [Solanum lycopersicum]
**Unigene0023558**	2.1	-2.6	bidirectional sugar transporter SWEET2a-like [Solanum lycopersicum]
**Unigene0041404**	4.2	-7.7	bidirectional sugar transporter NEC1-like [Solanum lycopersicum]
**Unigene0015567**	5.1	-5.3	peptide transporter PTR3-A-like [Fragariavesca subsp. vesca]
**Unigene0021742**	8.9	-7.0	Peptide transporter 1 isoform 1 [Theobroma cacao]
**Unigene0015568**	2.4	-2.4	peptide transporter PTR3-A-like [Solanum lycopersicum]
**Unigene0021745**	8.1	-8.2	Peptide transporter 1 isoform 1 [Theobroma cacao]
**Unigene0015566**	3.1	-3.7	peptide transporter PTR3-A-like [Cucumissativus]
**Unigene0023591**	5.1	-4.4	MATE efflux family protein 5 [Vitisvinifera]
**Unigene0014060**	3.1	-2.1	MATE efflux family protein [Theobroma cacao]
**Unigene0007370**	5.6	-7.9	MATE efflux family protein [Theobroma cacao]
**Unigene0023504**	6.0	-3.6	MATE efflux family protein [Theobroma cacao]
**Unigene0031906**	4.9	-3.5	MATE efflux family protein 8-like [Fragariavesca subsp. vesca]
**Unigene0018290**	4.4	-2.5	ABC transporter C family member 4-like [Solanum lycopersicum]
**Unigene0023528**	2.4	-4.8	ABC transporter B family member 21-like [Solanum lycopersicum]
**Unigene0035979**	11.7	-3.2	mutant histone deacetylase 6 [Arabidopsis thaliana]
**Unigene0006009**	2.1	-3.5	histone-lysine N-methyltransferase ASHR2-like isoform 1 [Solanum lycopersicum]
**Unigene0012248**	2.1	-3.3	Histone H3 K4-specific methyltransferase SET7/9 family protein [Theobroma cacao]
**Unigene0009759**	2.0	-3.9	Methyltransferases [Theobroma cacao]
**Unigene0013388**	2.1	-3.3	histone H1 [Solanum lycopersicum]
**Unigene0012434**	3.5	-7.9	Histone superfamily protein [Theobroma cacao]
**Unigene0007578**	2.1	-3.7	Histone H2B [Medicagotruncatula]
**Unigene0043003**	3.0	-4.6	PREDICTED: histone H2AX [Vitisvinifera]

To further identify the crucial regulatory genes, we investigated the protein kinases and the transcription factors in the 855 genes with functional descriptions. A total of 44 protein kinases were identified, which could be classified into 13 class-types. The top four classes were receptor-like protein kinase (11), L-type lectin-domain containing receptor kinase (6), LRR receptor-like serine/threonine-protein kinase (5), and leucine-rich repeat receptor-like protein kinase (5) (Table 4). A total of 38 transcription factors were identified, which could be categorized into eight TF families. Among these TF families, ERF (10), WRKY (8), and NAC (5) were the top three families with the most genes (Table 5).

**Table 4:** The putative kinase encoding genes that were induced by drought stress and repressed by re-watering.

Gene ID	Fold change (drought stress/control)	Fold change (re-watering/drought stress)	Blast swissprot
Unigene0020647	2.2	-3.7	receptor-like protein kinase HSL1-like [Solanum lycopersicum]
Unigene0020648	3.8	-2.9	receptor-like protein kinase HSL1-like [Cucumissativus]
Unigene0002856	2.0	-2.7	receptor-like protein kinase At5g47070-like [Vitisvinifera]
Unigene0001332	3.4	-3.6	receptor-like protein kinase HAIKU2-like [Solanum lycopersicum]
Unigene0008736	4.5	-2.0	receptor-like protein kinase At5g24010-like [Solanum lycopersicum]
Unigene0046009	6.1	-4.2	receptor-like protein kinase At1g11050 [Vitisvinifera]
Unigene0002857	3.6	-2.6	receptor-like protein kinase At5g47070-like [Vitisvinifera]
Unigene0023487	5.3	-4.6	receptor-like protein kinase HSL1-like [Vitisvinifera]
Unigene0007588	2.1	-2.3	receptor-like protein kinase At5g24010-like [Solanum lycopersicum]
Unigene0017838	3.1	-5.0	receptor-like protein kinase THESEUS 1-like [Vitisvinifera]
Unigene0022045	2.2	-2.4	receptor-like protein kinase At5g39020-like [Vitisvinifera]
Unigene0041990	4.1	-3.6	receptor protein kinase TMK1-like [Solanum lycopersicum]
Unigene0021193	2.8	-2.4	L-type lectin-domain containing receptor kinase S.4 [Vitisvinifera]
Unigene0000013	2.1	-4.7	L-type lectin-domain containing receptor kinase IX.1-like [Solanum lycopersicum]
Unigene0021196	2.6	-2.9	L-type lectin-domain containing receptor kinase S.4-like [Solanum lycopersicum]
Unigene0004102	3.0	-6.4	L-type lectin-domain containing receptor kinase S.5-like [Solanum lycopersicum]
Unigene0046764	17.7	-9.4	L-type lectin-domain containing receptor kinase VII.2-like [Glycine max]
Unigene0006242	2.7	-3.9	L-type lectin-domain containing receptor kinase S.1-like [Solanum lycopersicum]
Unigene0005992	4.4	-9.7	LRR receptor-like serine/threonine-protein kinase EFR-like [Fragariavesca subsp. vesca]
Unigene0020208	2.5	-2.2	LRR receptor-like serine/threonine-protein kinase At1g74360-like [Solanum lycopersicum]
Unigene0014178	2.0	-2.2	LRR receptor-like serine/threonine-protein kinase At2g16250-like [Fragariavesca subsp. vesca]
Unigene0015017	3.3	-3.1	LRR receptor-like serine/threonine-protein kinase At3g47570-like [Cucumissativus]
Unigene0019486	6.5	-5.6	LRR receptor-like serine/threonine-protein kinase GSO1-like [Solanum lycopersicum]
Unigene0020943	3.2	-3.1	leucine-rich repeat receptor-like protein kinase At2g33170-like [Vitisvinifera]
Unigene0020945	3.3	-4.2	leucine-rich repeat receptor-like protein kinase At2g33170-like [Solanum lycopersicum]
Unigene0008323	2.1	-2.3	leucine-rich repeat protein kinase family protein [Theobroma cacao]
Unigene0022012	3.9	-5.6	leucine-rich repeat receptor-like protein kinase At4g00330 [Vitisvinifera]
Unigene0020944	2.6	-3.3	leucine-rich repeat receptor-like protein kinase At2g33170-like [Solanum lycopersicum]
Unigene0010753	2.0	-3.5	adenylyl-sulfate kinase 1 [Vitisvinifera]
Unigene0005199	2.1	-2.3	acetylglutamate kinase, chloroplastic-like [Solanum lycopersicum]
Unigene0021288	2.4	-2.1	auxin-regulated dual specificity cytosolic kinase [Solanum lycopersicum]
Unigene0014244	2.1	-6.4	mitogen-activated protein kinase kinasekinase 3-like [Solanum lycopersicum]
Unigene0045145	9.0	-5.5	mitogen-activated protein kinase kinasekinase 2-like [Solanum lycopersicum]
Unigene0021384	2.9	-2.1	MAPKK [Nicotianatabacum]
Unigene0006235	3.1	-2.6	serine/threonine-protein kinase DDB_G0283821-like [Vitisvinifera]
Unigene0005255	16.6	-19.7	serine/threonine-protein kinase tsuA-like [Cicer arietinum]
Unigene0018210	4.7	-4.3	CBL-interacting serine/threonine-protein kinase 1-like [Fragariavesca subsp. vesca]
Unigene0021736	3.9	-3.5	cysteine-rich receptor-like protein kinase 42 [Vitisvinifera]
Unigene0021732	12.5	-45.4	cysteine-rich receptor-like protein kinase 42-like [Glycine max]
Unigene0024230	2.1	-2.4	G-type lectin S-receptor-like serine/threonine-protein kinase At4g27290-like [Vitisvinifera]
Unigene0006476	3.3	-3.4	G-type lectin S-receptor-like serine/threonine-protein kinase RKS1-like [Vitisvinifera]
Unigene0018279	3.0	-2.4	choline/ethanolamine kinase [Vitisvinifera]
Unigene0022845	16.2	-3.3	Concanavalin A-like lectin protein kinase family protein [Theobroma cacao]
Unigene0011240	2.6	-2.8	dual specificity protein kinase shkB [Vitisvinifera]

**Table 5:** The putative TFs genes that were induced by drought stress and repressed by re-watering.

Gene ID	Fold change (drought stress/control)	Fold change (re-watering/drought stress)	Blast swissprot
Unigene0042229	27.8	-13.9	Ethylene-responsive transcription factor ERF098-like [Solanum lycopersicum]
Unigene0004507	545.9	-8.9	Ethylene-responsive transcription factor ABR1-like [Solanum lycopersicum]
Unigene0023064	3.5	-22.2	Ethylene-responsive transcription factor ERF014-like [Cicer arietinum]
Unigene0020372	2.7	-2.6	Ethylene-responsive transcription factor ERF114-like [Vitisvinifera]
Unigene0025466	11.8	-142.9	Ethylene-responsive transcription factor ERF098-like [Solanum lycopersicum]
Unigene0003028	3.5	-12.1	Ethylene response factor 10 [Actinidiadeliciosa]
Unigene0002715	2.6	-6.6	Ethylene-responsive transcription factor 1A-like [Glycine max]
Unigene0023911	5.8	-5.9	Ethylene response factor 14 [Actinidiadeliciosa]
Unigene0003821	16.8	-6.7	Ethylene response factor 10 [Actinidiadeliciosa]
Unigene0017954	2.2	-4.8	Ethylene-responsive transcription factor RAP2-3 [Vitisvinifera]
Unigene0004183	2.5	-2.3	WRKY transcription factor 53 [Jatropha curcas]
Unigene0011852	3.4	-2.9	WRKY transcription factor 70-like [Vitisvinifera]
Unigene0003104	3.4	-2.2	WRKY transcription factor 48 [Vitisvinifera]
Unigene0003461	5.5	-3.3	WRKY22 [Catharanthusroseus]
Unigene0003103	2.2	-2.0	WRKY transcription factor 31 [(Populustomentosa x P. bolleana) x P. tomentosa]
Unigene0013608	3.0	-3.8	WRKY DNA-binding protein 27 [Arabidopsis thaliana]
Unigene0014567	2.1	-2.0	WRKY transcription factor 22-like [Solanum lycopersicum]
Unigene0017815	10.7	-5.5	WRKY transcription factor 75-like [Setariaitalica]
Unigene0012446	14.6	-3.6	NAC domain containing protein 80 [Theobroma cacao]
Unigene0021188	2.4	-3.1	NAC transcription factor [Camellia sinensis]
Unigene0002781	3.3	-3.2	NAC domain transcriptional regulator superfamily protein [Theobroma cacao]
Unigene0006055	5.6	-4.4	NAC transcription factor 29-like [Solanum lycopersicum]
Unigene0012447	11.0	-5.7	NAC domain-containing protein 100-like [Vitisvinifera]
Unigene0036849	2.3	-2.2	Myb-related protein 2 [Nicotianatabacum]
Unigene0030322	15.1	-21.9	Myb-related protein Myb4-like [Solanum lycopersicum]
Unigene0016812	4.2	-3.4	MYB1 [Gossypiumhirsutum]
Unigene0034565	4.3	-4.8	transcriptional activator Myb-like [Cucumissativus]
Unigene0022511	12.0	-4.1	Myb domain protein 112 isoform 1 [Theobroma cacao]
Unigene0000917	8.1	-5.6	transcription factor MYB75-like [Vitisvinifera]
Unigene0016518	3.6	-3.1	AP2/ERF domain-containing transcription factor [Populustrichocarpa]
Unigene0001595	6.5	-8.7	AP2/ERF domain-containing transcription factor [Populustrichocarpa]
Unigene0004302	9.8	-3.5	AP2/ERF domain-containing transcription factor [Populustrichocarpa]
Unigene0046101	32.2	-10.5	AP2/ERF domain-containing transcription factor [Populustrichocarpa]
Unigene0006534	3.1	-2.0	Basic helix-loop-helix DNA-binding family protein [Theobroma cacao]
Unigene0006504	3.9	-3.6	transcription factor bHLH96-like [Vitisvinifera]
Unigene0023715	7.8	-2.8	transcription factor bHLH74 [Vitisvinifera]
Unigene0009434	2.9	-2.2	BES1/BZR1-like protein [Medicagotruncatula]
Unigene0007439	5.7	-4.3	Trihelix transcription factor GT-3b-like [Cicer arietinum]

### Validation of the RNA-seq data by qRT-PCR

**Fig 7:**
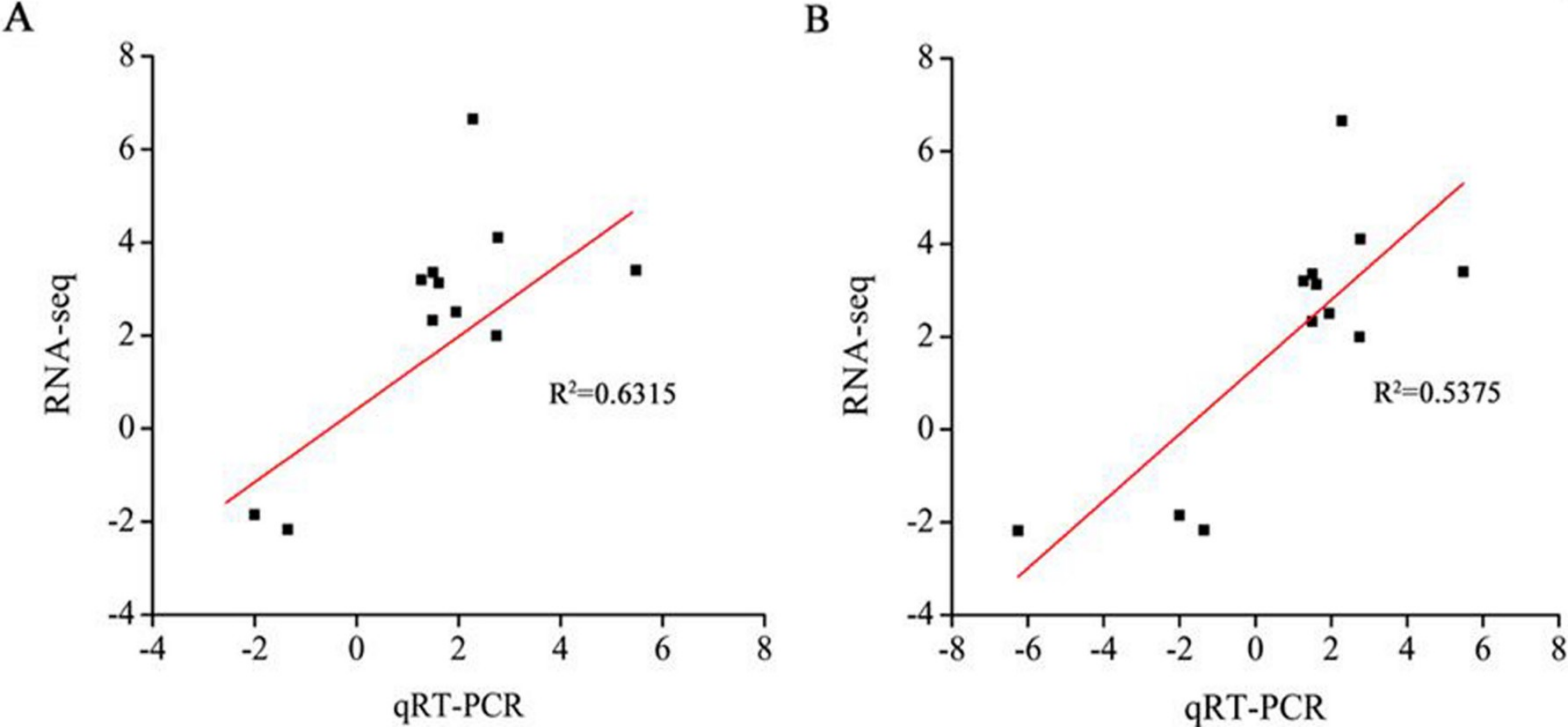
Verification of the differentially expressed genes in the control vs. drought stress group (A) and the control vs. re-watering group (B) by qRT-PCR.

To verify the validity of the RNA-seq data, we analyzed 24 genes using qRT-PCR (S10 Table). The correlation coefficients of the gene expression trends after qRT-PCR, and the sequencing data from control vs. drought stress and control vs. re-watering were 0.6315 and 0.5735, respectively (Fig 7), which confirmed the validity of the RNA-seq data.

## Discussion

In this study, we determined 5 physiological indexes of three groups. The results indicated that SOD, POD, proline, soluble sugar and MDA were increasing under drought stress. Then after re-watering all indexes were lower than drought stress, but they were significant higher than control. We performed a comparative analysis of the transcriptome changes of *M*. *tenacissima* undergoing drought stress and re-watering treatment. A total of 8672, 6043, 6537 DEGs were identified in drought stress vs control, re-watering vs control, and re-watering vs drought stress, respectively. Interestingly, there were 1174 up-regulated and 2344 down-regulated genes under drought stress presenting an opposite expression pattern when after re-watering, which were most possibly play important roles in tolerance to drought stress in *M*. *tenacissima*.

We found two aquaporin genes were up-regulated by drought stress and down-regulated after re-watering. This indicated that these aquaporin(s) may play a similar regulatory role in *M*. *tenacissima* under drought stress. The results have been confirmed in chickpea, foxtail millet, maize, and potato [37–40]. Heat shock responsive genes are functional genes that facilitate protein refolding and stabilize polypeptides and membranes. They have also been reported to respond to drought stress in barley, rice, and potato [32, 39–41]. In this study, we found that the expression of two heat shock responsive genes were induced by drought stress and repressed by re-watering treatment, which suggested that they directly participated in regulating drought-stress responses in *M*. *tenacissima*. Generally, drought stress induces the accumulation of LEA proteins and this accumulation enhances the survival rate of plants under drought conditions. The role of LEA proteins was to facilitate the correct folding of both structural and functional proteins and prevent lipid peroxidation [36, 42–46]. Previous research had showed that overexpression *IbLEA14* in sweet potato transgenic calli enhanced tolerance to drought and salt stress [47].In this study, the expression of three LEA encoding genes was significantly up-regulated by drought stress and down-regulated by re-watering treatment.

In this study, we found 44 putative kinases, which were up-regulated by drought stress and down-regulated after re-watering, that include receptor-like kinases(RLKs), L-type lectin-domain containing, serine/threonine-protein kinase, LRR receptor-like, MAPKK et al. RLKs play an important role in plant response to drought stress[48]. Overexpression of RLKs gene in transgenic rice that can enhance the tolerance to drought stress and salt [49].In our study, we found 11 RLKs genes up-regulated under drought stress. Mitogen-activated protein kinases (MAPK) pathways are known to be activated by numerous abiotic stresses such as cold, salt, heat, drought, ozone, or heavy metal intoxication [49]. Previous study had demonstrated that *PtrMAPK* acted as a positive regulator in dehydration/drought stress responses [49].

Trehalose has a protective role against various abiotic stresses, including drought stress in bacteria, fungi, and some plants. It helps maintain cellular membrane integrity and prevent protein degradation [50–51]. In plants, trehalose-6-phosphate synthase (TPS) and trehalose-6-phosphate phosphatase catalyze the biosynthesis of trehalose, and their expressions are induced by drought stress [36, 52–54]. Furthermore, overexpression *AtTPS1* or *OsTTPS1* improved the stress resistance of transgenic plants [52–54]. In this study, one TPP gene and two TPS encoding genes were up-regulated by drought stress and down-regulated after re-watering. Furthermore, one proline synthesis-related gene had a similar expression pattern to the TPP and TPS genes. These results indicate that synthesizing compatible solutes is a conserved drought resistance mechanism in different plants.

It’s well-known that drought stress produces reactive oxygen species (ROS) and excessive ROS can cause the irreversible oxidization of lipids and proteins, which leads to membrane injury [55]. To overcome ROS injury, plants utilize ROS-scavenging enzymes, such as peroxidase (POD), superoxide dismutase (SOD), and catalase (CAT), to scavenge the excessive ROS [56]. Our physiological results showed the activity of SOD and POD were doubled under drought stress. We also found six POD-encoding genes were significantly up-regulated by drought stress and down-regulated by re-watering treatment in the transcriptome data, which indicated that ROS-scavenging via POD is an important mechanism in the overall resistance of *M*. *tenacissima* to drought stress.

Some studies have shown that alcohol dehydrogenase, MATE efflux family protein, and ABC transporters are involved in protecting plants against drought stress [57–61]. In this study, three alcohol dehydrogenase-, five MATE-, and two ABC-encoding genes were identified, and their expressions were strongly induced by drought stress, but significantly repressed by re-watering treatment, which suggested that these genes may also play important roles in drought stress resistance.

We identified 38 TFs that were significantly up-regulated by drought stress and down-regulated after re-watering. The 38 TFs could be classified into the AP2/ERF, bHLH, BES1, ERF, MYB, MYB-related, NAC, WRKY, and Trihelix families. Among them, the ERF (10), WRKY (8), NAC (5), and AP2/ERF (4) families accounted for 70% of the genes, which acted as key regulators and play crucial roles in plant resistance to drought stress [12–17].AP2/EREBPs, WRKYs, and NACs were key regulators of ABA-mediated stomatal closure [62–64]. bHLHs play an important role in the JA-mediated regulatory network of the abiotic stress response[65].This indicated that ABA and JA play a central role in regulating drought stress tolerance in *M*. *tenacissima*.

## Conclusion

The activity of SOD and POD were doubled under drought stress. In this study, we performed a comparative analysis of the transcriptome changes in *M*. *tenacissima* undergoing drought stress and re-watering treatment. A total of 8672, 6043, and 6537 DEGs, including 363, 267, and 299 TFs, were identified in the control vs. drought stress, control vs. re-watering, and drought stress vs. re-watering comparisons, respectively. The DEGs from these three comparative groups were classified into 67, 58, and 66 GO categories and were involved in 120, 114, and 115 KEGG pathways, respectively. Interestingly, 1174 up-regulated and 2344 down-regulated genes under drought stress had the opposite expression pattern after re-watering. Analysis of the 1174 up-regulated genes induced by drought stress and repressed by re-watering showed that many genes were homologous to known functional genes that directly protect plants against drought stress. Furthermore, 44 protein kinases and 38 TFs with opposite expression patterns under drought stress and re-watering were identified as crucial candidate regulators of drought stress resistance in *M*. *tenacissima*.

In summary, our study is the first to characterize the *M*. *tenacissima* transcriptome in response to drought stress, and has identified the key candidate drought stress resistant genes in *M*. *tenacissima*. Our results will help unravel the mechanism controlling *M*. *tenacissima* drought stress resistance.

## Supporting information

S1 Table Differentially expressed genes that were up-regulated in the control vs. drought stress comparison and down-regulated in the drought stress vs. re-watering comparison. (XLSX)

S2 Table Differentially expressed genes that were down-regulated in the control vs. drought stress comparison and up-regulated in the drought stress vs. re-watering comparison. (XLSX)

S3 Table Significantly differentially expressed genes identified in the control vs. drought stress comparison that were annotated to KEGG pathways. (XLSX)

S4 Table Significantly differentially expressed genes identified in the control vs. re-watering comparison that were annotated to KEGG pathways. (XLSX)

S5 Table Significantly differentially expressed genes identified in the drought stress vs. re-watering comparison that were annotated to KEGG pathways. (XLSX)

S6 Table Differentially expressed transcription factors identified in the control vs. drought stress comparison. (XLSX)

S7 Table Differentially expressed transcription factors identified in the control vs. re-watering comparison. (XLSX)

S8 Table Differentially expressed transcription factors identified in the drought stress vs. re-watering comparison. (XLSX)

S9 Table Genes that were up-regulated under drought stress and down-regulated after re-watering treatment. (XLSX)

S10 Table Log2 fold change data for the RNA-seq and qRT-PCR analyses. (XLSX)
